# Discoveries Interview: Professor Derek J. Hausenloy on the ischemic heart disease

**DOI:** 10.15190/d.2015.35

**Published:** 2015-04-08

**Keywords:** professor Derek Hausenloy, interview

**Figure 1:**
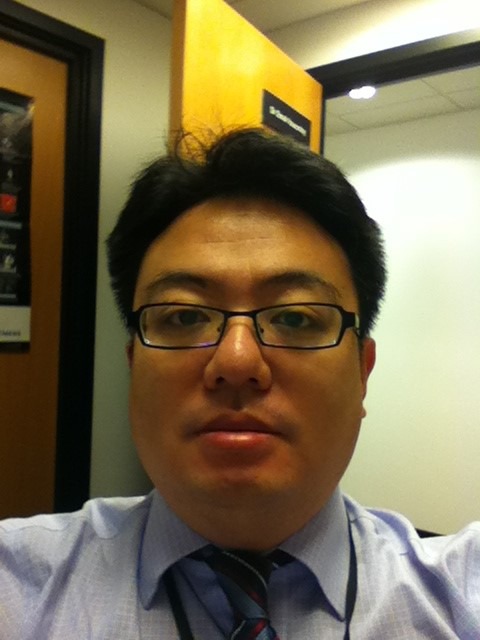
Professor Derek J. Hausenloy

**Dr. Derek J. Hausenloy** is a Professor at Duke-National University of Singapore and University College London.

He is Honorary Consultant Cardiologist at the National Heart Centre Singapore and the Barts Heart Centre.

Professor Hausenloy graduated from Manchester University, UK, and performed a PhD in Cardiovascular Medicine at the University College London, UK, investigating the role of mitochondria as targets for cardioprotection. He performed his clinical Cardiology training in London and subspecialized in cardiac MRI. His main research interests include cardioprotection (pre-clinical experimental studies, clinical proof-of-concept clinical studies, and multicentre clinical outcome studies) and small animal and clinical cardiac magnetic resonance imaging, publishing numerous findings on the subject.

He is also a Fellow of the European Society of Cardiology (FESC) and American College of Cardiology (FACC).

## 1. Can you define in simple words the ischemic heart disease and why studying it is so important?

Ischemic heart disease is the leading cause of death and disability worldwide. One major clinical manifestation of ischemic heart disease is an acute myocardial infarction. For patients presenting with an acute myocardial infarction, a blockage within the coronary artery deprives oxygen and nutrients to the myocardium resulting in damage to the heart muscle (termed ischemic injury). In these patients, the treatment of choice is to remove the blockage using coronary angioplasty to restore blood flow to the heart muscle - paradoxically, this can in itself cause further damage to the heart (termed reperfusion injury)^1^. By understanding the pathophysiology underlying the effect of ischemia/reperfusion injury on the heart, we hope to identify new treatments for protecting the heart, thereby preserving cardiac contractile function and improving clinical outcomes in patients with ischemic heart disease.

## 2. How our knowledge on ischemia/reperfusion injury evolved over the time?

Initial research in this field focused on the detrimental effects of ischemia on the myocardium, but over the time, it has become apparent that, although reperfusion is essential to salvage viable heart muscle following an acute myocardial infarction, restoring blood flow can in itself damage the heart muscle – termed myocardial reperfusion injury^1,2^. Crucially, although treatments are in place to limit ischemic injury, there is currently no effective therapy for preventing reperfusion injury, and so new treatments are required to target and prevent this form of myocardial injury.

## 3. Why is translation of knowledge into clinical practice so challenging?

Although a vast number of new treatments for protecting the heart against ischemia/reperfusion injury have been discovered in the research laboratory, the translation of this knowledge into the clinical setting has been challenging. The reasons for this are many and include the use of animal ischemia/reperfusion models, which do not accurately represent the patient presenting with an acute myocardial infarction, and the inadequate design of the clinical studies^3^.

## 4. Which treatments have the potential to protect the heart muscle from ischemia/ reperfusion injury?

By subjecting the heart to brief non-lethal episodes of ischemia and reperfusion it is possible to upregulate endogenous mechanisms of protection within the heart and render the myocardium resistant to the detrimental effects of ischemia/reperfusion injury – a phenomenon which has been termed ‘ischemic conditioning’. The most promising approach to translating ischemic conditioning into the clinical setting is to apply the protective stimulus to the arm by simply inflating and deflating a blood pressure cuff placed on the upper arm – a phenomenon termed ‘remote ischemic conditioning’^4^. This treatment strategy has been shown to be effective at preventing ischemia/reperfusion injury in patients presenting with an acute myocardial infarction (with a 27% reduction in myocardial infarct size)^5^ and in patients undergoing cardiac bypass surgery (with a 43% reduction in peri-operative myocardial injury)^6^.

## 5. What will the field look like in 5-10 years?

In 5-10 years, I would hope that we will have discovered a new treatment for protecting the heart against ischemia/reperfusion injury which is capable of improving clinical outcomes in acute myocardial infarction patients, in terms of improved patient survival and less heart failure.

## 6. What advice do you have for young scientists?

Given the challenges of pursuing a career in research, it is essential that the young scientist be passionate for research and have a good mentor looking out for them.
